# Clinician and Patient Perspectives of Geographic Atrophy Age-Related Macular Degeneration in Spain: A Preliminary Exploratory Study

**DOI:** 10.3390/jcm15145596

**Published:** 2026-07-16

**Authors:** David P. Piñero, Laurent Bataille, Julio Cesar Molina Martín, Rafael J. Pérez-Cambrodí

**Affiliations:** 1 Department of Optics, Pharmacology and Anatomy, University of Alicante, 03690 San Vicente del Raspeig, Alicante, Spain; rjcambrodi@gmail.com; 2 Department of Ophthalmology, Vithas Medimar International Hospital, 03016 Alicante, Alicante, Spain; 3 Visitrain S.L., Science Park, University of Alicante, 03005 Alicante, Alicante, Spain; lbataille@visitraintherapies.com; 4 Servicio de Oftalmología, Hospital Universitario San Juan de Alicante, 03550 Sant Joan d’Alacant, Alicante, Spain; jcmm.molina@gmail.com

**Keywords:** geographic atrophy, low vision, age-related macular degeneration, visual rehabilitation

## Abstract

**Background/Objectives**: We aim to characterize, through a survey-based approach conducted in Spain, the perspectives of healthcare professionals (HCPs) and patients regarding geographic atrophy (GA). **Methods**: A survey-based study assessed ophthalmologists’, optometrists’, and AMD patients’ perceptions and practices regarding geographic atrophy management in Spain, including disease journey challenges. **Results**: The study sample comprised 30 ophthalmologists, 35 optometrists, and 37 patients. A substantial majority of ophthalmologists followed a specific protocol for the management of GA, in which optical coherence tomography, visual acuity assessment, and fundus examination constituted the core diagnostic procedures. Although most HCPs reported recommending low vision aids, only 5.4% of patients reported having received such a recommendation or prescription (*p* < 0.001). Considerable variability among the optometrists surveyed was identified regarding visual rehabilitation programmes involving low vision aids. Furthermore, while most ophthalmologists and optometrists reported having provided various types of disease-related information, a significantly lower proportion of patients reported having received information on several aspects such as the disease itself (*p* < 0.001), disease progression (*p* < 0.001), impact on quality of life (*p* < 0.001), or low vision aids (*p* < 0.001). Multiple limitations in daily living activities were reported by patients, with those diagnosed with GA experiencing significantly greater difficulty reading television subtitles (*p* = 0.026) and performing manual tasks (*p* = 0.005). **Conclusions**: HCPs appear to follow specific protocols and guidelines in the management of GA; however, a potential deficit in effective HCP–patient communication has been identified that should be investigated further.

## 1. Introduction

Geographic atrophy (GA) represents an advanced stage of age-related macular degeneration (AMD), characterized by progressive and permanent impairment of vision [1,2]. The condition is marked by well-defined areas of atrophy in the outer retina, caused by the degeneration of photoreceptors, retinal pigment epithelium (RPE), and the underlying choriocapillaris [1,2]. These lesions usually emerge near the perifoveal macula, often leaving the foveal center unaffected at first; with time, they tend to grow and merge, eventually involving the fovea [1,2].

Although GA progression differs considerably between patients, growing research suggests that specific characteristics may be useful in predicting how the condition develops and what results can be anticipated [3,4]. A number of factors that may help determine an individual’s disease outlook have been consistently confirmed across multiple patient groups: the initial size of the lesion, its location, whether multiple lesions are present, fundus autofluorescense (FAF) patterns, and the condition of the other eye. Since best-corrected visual acuity does not directly reflect GA lesion growth due to the possibility of the fovea being spared, alternative measures haven been used to investigate the relationship between structural progression and the decline of visual function, including microperimetry, low-luminance visual acuity, or reading speed tests [3,4].

This retinal disorder represents a leading etiology of visual impairment among the elderly population and is associated with a substantial decline in functional capacity [5]. The most frequently reported symptom is difficulty engaging in activities of daily living, especially reading and near-vision tasks [5]. These visual limitations have been shown to elicit varying degrees of fear, frustration, and anxiety, all of which may profoundly compromise patients’ quality of life [5,6,7]. A multinational survey conducted among individuals aged 60 years and older with a self-reported diagnosis of geographic atrophy (GA), residing in the United States, Canada, Australia, and six European countries, revealed that the majority of respondents (77%) perceived GA as progressing more rapidly than anticipated, while 68% reported an inability to fully enjoy life as they had prior to diagnosis [6]. Regarding psychosocial outcomes, 49% of participants with bilateral GA and 56% of those with unilateral GA reported moderate-to-major negative effects on self-confidence, whereas 40% of both cohorts described moderate-to-major adverse impacts on mental health [6].

Although considerable consensus exists among ophthalmologists, optometrists, and visual rehabilitation specialists regarding multiple aspects of GA management, incorporating the patient’s perspective remains paramount in order to deliver individualized care and minimize the disease’s impact on the patient’s visual quality of life [8]. The aim of the present study was to characterize, through a survey-based approach conducted in Spain, the perspectives of ophthalmologists, optometrists, and patients regarding GA, in order to provide a comprehensive overview of the diagnostic, therapeutic and visual rehabilitation processes and to better understand patients’ symptoms, concerns, and unmet needs.

## 2. Materials and Methods

### 2.1. Participants

A cross-sectional, survey-based study was designed to assess the perceptions and practices regarding the management of geographic atrophy (GA) among ophthalmologists (retina specialists exclusively) and optometrists (low vision specialists exclusively) in Spain, as well as the GA patients’ perspective in terms of their disease journey and the challenges and difficulties associated with this condition. The study was conducted in accordance with the principles outlined in the Declaration of Helsinki and received approval from the Ethics Committee of the University of Alicante.

Data collection was carried out through an online questionnaire administered via Google Forms (Google, Mountain View, CA, USA). Three distinct versions were developed: one for retinal specialists, one for optometrists specializing in low vision, and a third for patients diagnosed with GA. Prior to initiating the survey, participants were required to provide a Gmail address, thereby preventing the submission of duplicate responses from the same individual. Eligible participants included ophthalmologists and optometrists practicing in Spain. To this end, members of the Sociedad Española de Retina y Vítreo (SERV) and the Sociedad Española de Especialistas en Baja Visión (SEEBV) were invited to participate in the study. Patients were recruited through members of the following patient associations: Baja Visión 3C, Acción Visión España, and Asociación AMIRES, as well as from patients attending the Retina Unit of the Department of Ophthalmology at Hospital Universitario San Juan de Alicante.

### 2.2. Characteristics of the Online Surveys

The online surveys were developed by one ophthalmologist and one optometrist specializing in low vision according to their own clinical experience of more than 20 years. Each survey consisted of four to five sections with a variable number of questions, as detailed in Table 1. All surveys were administered exclusively in Spanish. The questionnaires targeting ophthalmologists and optometrists were distributed via email and completed between 15 March and 15 June 2025. During the same period, the patient survey was disseminated via email through patient associations. An exception was made for patients attending the Retina Unit of the Hospital Universitario San Juan de Alicante, for whom a paper-based version of the survey was administered during scheduled consultations.

All three surveys included an introductory section providing information about the study and requesting participants’ informed consent. Data collection was contingent upon participants actively confirming their consent in the first question. Following this, the first section gathered demographic information from both professionals and patients, encompassing variables such as age, sex, years of professional experience, and current workplace setting. The second section, directed at ophthalmologists and optometrists, addressed clinical practice with geographic atrophy (GA) patients, with questions tailored to the specific roles and functions of each professional group. For patients, the second section assessed their knowledge of AMD and their patient journey. The third section explored the prescription and recommendation of low vision aids among ophthalmologists and optometrists, as well as their use by patients. The fourth section varied across surveys: it covered telescopic intraocular lenses (IOLs) for ophthalmologists, visual rehabilitation with low vision aids for optometrists, and information and clinical support received for patients. Finally, a fifth section, included exclusively in the optometrists’ survey, assessed their knowledge of telescopic IOLs.

Prior to administering the survey to the final study population, it was piloted with 10 ophthalmologists, 10 optometrists, and 10 patients to confirm that all questions were clearly understood. These participants were not subsequently included in the study described here. This preliminary exploration allowed the research team to refine the wording of certain questions in order to avoid confusion. No further reliability assessments were performed.

### 2.3. Data Analysis

Statistical analyses were performed using the Statistical Package for the Social Sciences, version 26.0 (SPSS Inc., Chicago, IL, USA). Differences in the perception of selected survey aspects between optometrists and ophthalmologists, as well as between healthcare professionals and patients, were assessed using the Chi-square test. When more than 20% of cells presented expected frequencies below 5, Fisher’s exact test was applied as an alternative. As multiple comparisons were performed, the Bonferroni correction was applied to adjust the level of statistical significance. Statistical significance was set at *p* < 0.05. There were no missing data, and no adjustment was required regarding this issue.

## 3. Results

The study sample comprised three groups: 30 ophthalmologists (29.4%), 35 optometrists (34.3%), and 37 patients (36.3%), totaling 102 participants. The principal findings for each group are presented in the subsequent sections.

### 3.1. Results Group Ophthalmologists

The ophthalmologists surveyed were highly experienced professionals: 28 (93.3%) had more than 10 years of experience and 2 (6.7%) had between 6 and 9 years of experience. The majority were male (22, 73.3%). Regarding workplace distribution, most worked in public hospitals (18, 60.0%), followed by private hospitals (8, 26.7%), private ophthalmological practices (3, 10.0%), and university clinics (1, 3.3%). A substantial majority of ophthalmologists reported having a specific protocol for the management of patients with GA (22, 73.3%).

Regarding the clinical tests considered essential in the management of GA patients, the vast majority of ophthalmologists surveyed agreed on the use of optical coherence tomography (OCT) (30, 100%), assessment of visual acuity (29, 96.7%), refraction (20, 66.7%), and fundus examination (27, 90.0%). Fundus autofluorescence (FAF) was identified as a relevant clinical test in the management of GA by 33.3% of the ophthalmologists surveyed.

**Figure 1:**
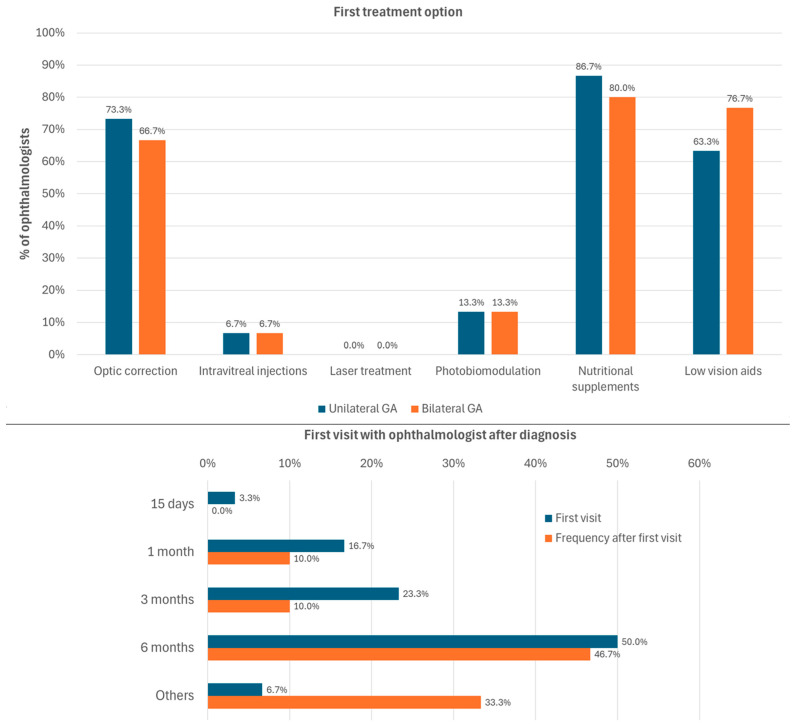
Treatment recommendations for geographic atrophy (GA) among the ophthalmologists surveyed (**top**) and distribution of the first follow-up visit scheduled following diagnosis (**bottom**).

Treatment recommendations are summarized in Figure 1. Among the first-line treatment options prescribed by ophthalmologists for GA, optical correction, nutritional supplements, and low vision aids were the most frequently reported. A trend towards a lower prescription rate of nutritional supplements (86.7% vs. 80.0%) and a higher prescription rate of low vision aids (63.3% vs. 76.7%) was observed in cases of bilateral GA. Regarding the first follow-up visit after diagnosis, the majority of ophthalmologists agreed on scheduling it at 3 months (23.3%) or 6 months (50.0%), with subsequent visits conducted every 6 months (46.7%) or 12 months (33.3%) in most cases. Concerning cataract surgery, the most frequently implanted intraocular lens (IOL) was the monofocal lens, with 6.7% of ophthalmologists using extended depth of focus (EDOF) IOLs in unilateral GA and 23.3% using telescopic IOLs in bilateral GA. The majority of postoperative protocols reported by the ophthalmologists surveyed included follow-up visits at 1 day, 1 month, and 3 months after surgery.

**Figure 2:**
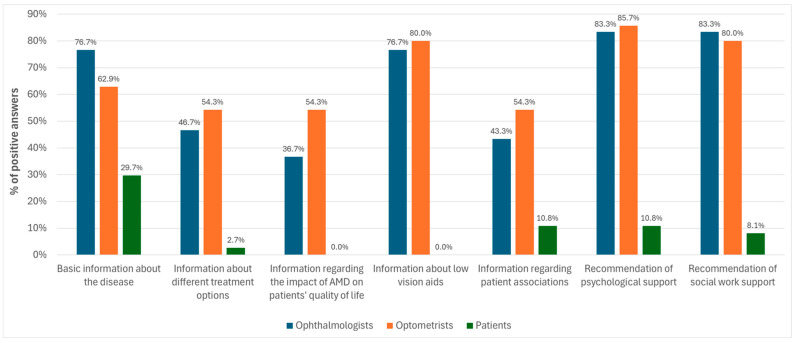
Distribution of information on various aspects of age-related macular degeneration provided by ophthalmologists and optometrists, and patients’ perception of the information received regarding those aspects.

Another relevant aspect concerns the information provided by ophthalmologists for patients (Figure 2). The majority agreed on providing basic information about the disease (76.7%) and information about low vision aids (76.7%), as well as recommending psychological and social work support (83.3%). The vast majority of ophthalmologists (27, 90.0%) reported recommending low vision aids to improve visual performance.

### 3.2. Results Group Optometrists

The optometrists surveyed were also highly experienced professionals: 33 (94.3%) had more than 10 years of experience and 2 (5.7%) had between 6 and 9 years of experience. The sample was relatively balanced in terms of sex distribution, with 20 males (57.1%) and 15 females (42.9%). Regarding workplace distribution, the majority worked in private optometric practices (20, 57.1%), followed by public hospitals (5, 14.3%), university clinics (5, 14.3%), private hospitals (4, 11.4%), and private ophthalmological practices (1, 2.9%). A substantial majority of optometrists reported following a specific protocol for the management of patients with GA, which was available in the institution they work (27, 77.1%).

Optometrists were asked about the activities in which they were involved during the evaluation of GA patients, with the majority reporting the performance of anamnesis (30, 85.7%), assessment of visual acuity and refraction (33, 94.3%), Amsler grid evaluation (25, 71.4%), assessment of disease impact using validated questionnaires (17, 48.6%), contrast sensitivity evaluation (22, 62.9%), color vision assessment (16, 45.7%), and fundus screening by retinography (25, 71.4%). A total of 30 optometrists (85.7%) reported providing some form of information to patients. Another relevant aspect concerns the information provided by optometrists to patients (Figure 2), with the majority agreeing on providing basic information about the disease (62.9%) and information about low vision aids (80.0%), as well as recommending psychological (85.7%) and social work support (80.0%).

All optometrists reported recommending low vision aids to improve visual performance. Of these, 71.4% (25) routinely prescribed low vision aids in their clinical practice, with the majority reporting having managed low vision aid prescription in more than 20 patients (20, 57.1%). Regarding the timing of prescription, most optometrists preferred to prescribe once medical treatment had been completed (10, 40.0%), or immediately upon detection of the condition (36.0%). Only 4 optometrists (16.0%) reported prescribing upon specific indication from an ophthalmologist, while 2 optometrists (8.0%) did not report a specific criterion regarding this aspect.

The following aids were reported as commonly prescribed by optometrists working with low vision patients: magnifying glasses (24, 96.0%), recommendations for the use of zoom and text resizing features in digital devices (mobile and desktop) (24, 96.0%), telescopes (18, 72.0%), telemicroscopes (14, 56.0%), microscopes (21, 84.0%), selective absorption filters (24, 96.0%), virtual reality aids (10, 40.0%), augmented reality smart glasses (12, 48.0%), closed-circuit television (CCTV) (14, 56.0%), and non-optical aids (14, 56.0%).

Of the optometrists prescribing low vision aids, 72.0% (18) recommended a visual rehabilitation program conducted by themselves. Furthermore, 4 optometrists (16.0%) recommended visual rehabilitation but referred patients to other specialists, 2 (8.0%) reported recommending visual rehabilitation with low vision aids performed by themselves only on an occasional basis, and 1 (4.0%) indicated that such programs are not necessary. All optometrists practicing visual rehabilitation with low vision aids reported conducting in-office training sessions combined with home-based visual training.

**Figure 3:**
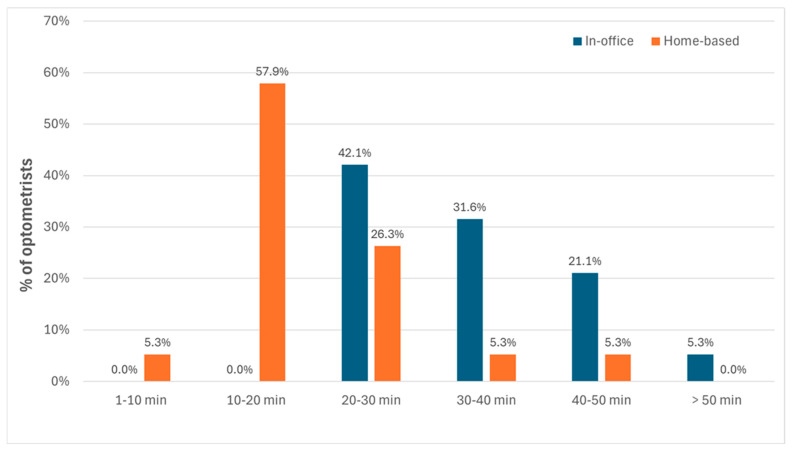
Distribution of the prescribed session duration for in-office and home-based visual rehabilitation with low vision aids among optometrists performing this type of training.

The duration of the visual rehabilitation program varied considerably among professionals: 1 to 15 days (3, 15.0%), 15 to 30 days (4, 20.0%), 30 to 60 days (5, 25.0%), 60 to 80 days (1, 5.0%), more than 80 days (1, 5.0%), and customized according to the individual patient’s capacities (6, 30.0%). As shown in Figure 3, the majority of optometrists reported conducting in-office visual rehabilitation sessions lasting between 20 and 40 min, while most also prescribed home-based sessions of between 10 and 30 min. The following distribution of training techniques was reported: use of fixation process re-education techniques (18, 90.0%), training of compensatory saccadic eye movements (16, 80.0%), stimulation through restoration techniques based on Gabor sinusoidal gratings or other types of visual stimuli (4, 20.0%), and visual training with virtual reality (7, 35.0%).

Finally, the majority of the optometrists surveyed considered that the field of prescription and fitting of low vision aids in AMD lacks sufficient recognition and professional prestige (25, 71.4%). According to these professionals, the main reasons attributed to this situation were the high cost of these services (18, 52.9%) and the absence of adequately trained professionals dedicated to this field (21, 61.8%). Additional contributory factors reported included: the absence of clearly defined clinical indications (6, 17.6%), insufficient scientific evidence supporting the field (5, 14.7%), and poor professional reputation or perception of the discipline as a placebo therapy (6, 17.6%).

### 3.3. Results Group Patients

The mean age of the patients surveyed was 68.1 years (Standard deviation, SD: 12.7; median: 71.5; range: 53–89 years). The sample was relatively balanced in terms of sex distribution, with 16 males (43.2%) and 21 females (56.8%). The majority of patients reported being retired (23, 62.2%), although other employment situations were also recorded: active but on leave (2, 5.4%), actively employed (7, 18.9%), unemployed (1, 2.7%), and disabled (4, 10.8%). Most participants resided in an urban area (34, 91.9%), with only 3 (8.1%) reporting residence in a rural area.

**Figure 4:**
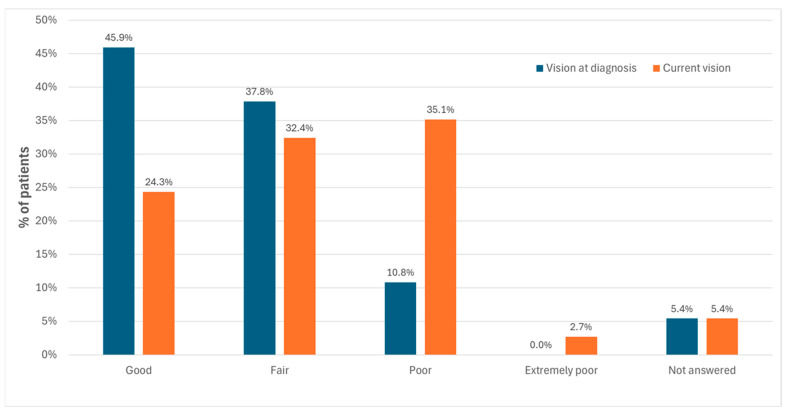
Distribution of responses regarding patients’ self-reported level of vision at the current time and at the time of diagnosis.

Regarding the type of AMD, 12 patients reported having neovascular AMD (32.4%), 13 reported having GA (35.1%), and 12 (32.4%) were uncertain about their specific type of AMD. In most cases, both eyes were affected (23, 62.2%), with only 10 patients (27.0%) reporting involvement of the right eye only and 4 (10.8%) reporting involvement of the left eye only. At the time of diagnosis, 45.9% of patients reported good visual acuity, whereas at the time of the survey only 24.3% reported the same level of vision (Figure 4). Notably, 35.1% of patients reported currently experiencing poor vision.

**Figure 5:**
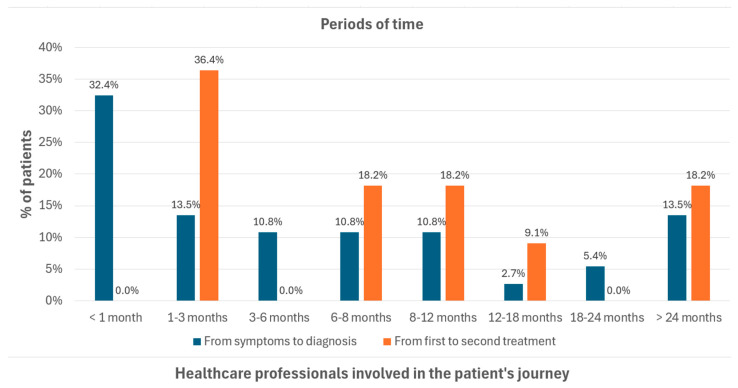
Distribution of responses regarding time intervals between symptom onset and diagnosis, and between first- and second-line treatments where applicable according to patients.

**Figure 6:**
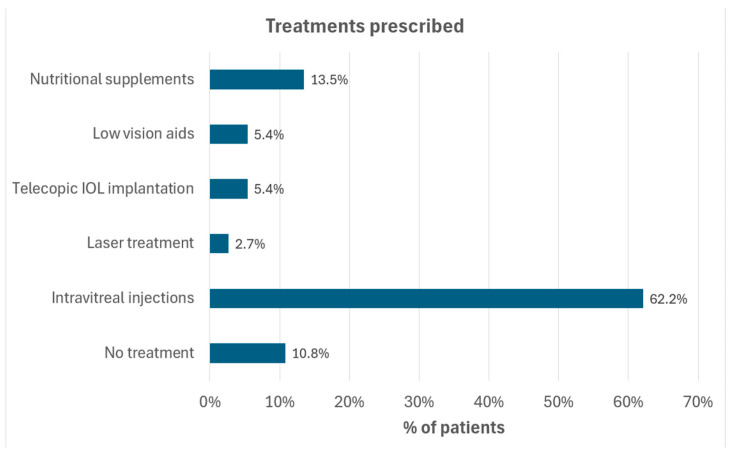
Distribution of responses regarding pharmacological and non-pharmacological treatments prescribed according to patients.

The majority of surveyed patients achieved a relatively prompt diagnosis, with 78.3% reporting a symptom-to-diagnosis interval ranging from under one month to twelve months (Figure 5). Regarding the first healthcare professional to evaluate the patient, an equal distribution was observed between ophthalmologists practicing in public (43.2%) and private institutions (43.2%). Nevertheless, diagnosis was confirmed more frequently by ophthalmologists in the public sector (56.8%), who also assumed the predominant role in long-term follow-up (89.2%). The most frequently prescribed treatment was intravitreal injections (62.2%), followed by nutritional supplements (13.5%). Notably, 40.5% of patients (15) reported having received ten or more injections (Figure 6). Despite these interventions, a substantial proportion of patients (14; 37.8%) reported no clinical improvement. In a subgroup of 11 patients (29.7%), a change of treatment was warranted, primarily due to the progression of visual loss (6) or continued retinal deterioration (2). Overall, 17 patients (45.9%) reported being dissatisfied with the outcomes of their treatment, primarily due to the absence of perceived clinical improvement.

Patients reported that the information provided was insufficient. Only 29.7% reported having received basic information about the disease, and 10.8% reported having received information about patient associations (Figure 4). Similarly, the majority of patients had not received recommendations for psychological support (10.8%) or social work support (8.1%). Only seven patients reported having received information about telescopic intraocular lenses (IOLs) (18.9%). Table 2 summarizes the symptoms, limitations, and difficulties in activities of daily living reported by patients, as well as the recommendations received from healthcare professionals. The most commonly reported symptoms were difficulty in reading (73.0%), followed by generalized blurred vision (48.6%). Similarly, a large proportion of patients reported the following difficulties in activities of daily living: reading product prices when shopping (67.6%), reading subtitles on television (67.6%), and reading newspaper text (64.9%). Regarding the recommendations received from healthcare professionals, the majority of patients had received two general recommendations: consistent use of sunglasses in sun-exposed environments (83.8%) and monitoring macular status by means of a grid-based self-assessment tool (known as the Amsler grid) (64.9%).

**Figure 7:**
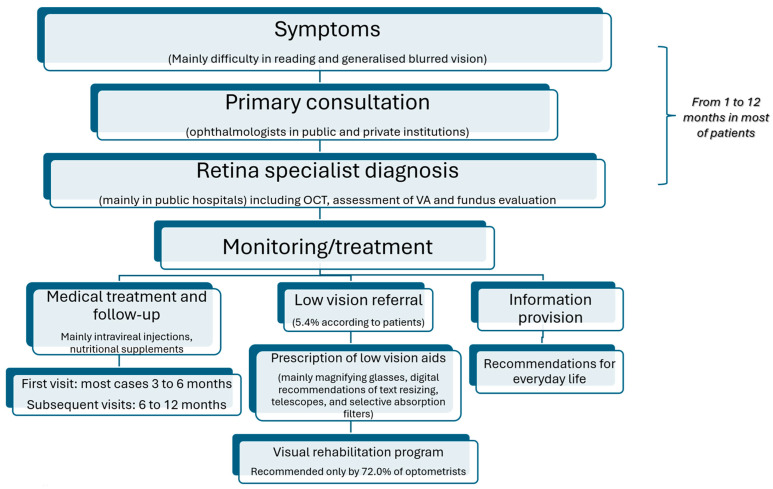
Preliminary Patient Journey for Geographic Atrophy in Spain.

Integrating the patient’s perspective with the protocols established by healthcare professionals, a proposed patient journey has been developed, as illustrated in Figure 7. This figure synthesizes and summarizes the responses collected across all questions included in the questionnaire administered throughout the present preliminary exploratory study, integrating the full range of information gathered from the participating ophthalmologists, optometrists, and patients. By consolidating these responses into a single comprehensive framework, the figure offers a flexible structure that is adaptable to individual patient variations and clinical particularities, while also accommodating the diversity of perspectives captured across the different professional and patient groups surveyed. In doing so, it thereby accurately reflects the real-world burden and management of the disease within the Spanish healthcare context, providing a holistic visual representation that goes beyond isolated data points to capture the broader patterns and relationships emerging from the study as a whole. 

### 3.4. Comparison Between Groups

When comparing the information reported as provided by ophthalmologists and optometrists with that reported as received by patients, significant differences were detected. Whereas the majority of ophthalmologists and optometrists reported having provided various types of information, a significantly lower proportion of patients reported having received such information, including: basic information about the disease (*p* < 0.001), disease progression (*p* < 0.001), treatment options (*p* < 0.001), impact on quality of life (*p* < 0.001), low vision aids (*p* < 0.001), and patient associations (*p* = 0.003). Furthermore, while the majority of ophthalmologists (unilateral GA: 63.3%; bilateral GA: 76.7%) and optometrists (100%) reported recommending or prescribing low vision aids, a significantly lower proportion of patients (5.4%) reported having received such a recommendation or prescription (*p* < 0.001).

Regarding patient-reported assessments, no statistically significant differences were found among patients with neovascular AMD, GA, or unspecified AMD in the self-evaluation of visual function at diagnosis (*p* = 0.179) or at the time of completing the survey (*p* = 0.318). No significant differences in symptom profiles were identified across AMD subtypes (*p* ≥ 0.093), with the exception of glare sensation, which was reported significantly more frequently in the GA group (*p* = 0.007). Similarly, no significant differences were observed among AMD subtypes across various aspects of the patient journey, including time intervals, healthcare professionals involved in follow-up, and perceived improvement with treatment (*p* ≥ 0.075). The only significant intergroup difference identified was a higher frequency of reported intravitreal injection use in the neovascular AMD group (*p* = 0.045). No significant differences between AMD subgroups were found with respect to information received or recommendations provided (*p* ≥ 0.186). However, GA patients reported significantly greater difficulty with reading subtitles on television (*p* = 0.026) and performing manual tasks (*p* = 0.005).

## 4. Discussion

This study was designed to provide a cross-sectional overview of the diagnostic, therapeutic, and follow-up processes in patients with geographic atrophy (GA), with the aim of obtaining a comprehensive understanding of the clinical experience of this patient population. The specific experience of Spain is presented, highlighting the need to investigate differences across healthcare settings, given that the healthcare conditions of each country are crucial, as are the healthcare professionals involved. One of the most relevant findings of this study is the lack of concordance between healthcare professionals’ perceptions of the information they provide to patients and patients’ actual perceptions of the information they receive. Furthermore, the pivotal role of ophthalmologists in public hospitals in confirming the diagnosis of GA and monitoring disease progression is clearly shown. The importance of diagnostic tests such as visual acuity measurement, refraction, and fundus evaluation including OCT analysis is also highlighted. Patient concern regarding the lack of improvement with current treatments is equally evident, which may be related to an insufficient understanding of the disease. This comprehensive cross-sectional overview enables the re-evaluation of certain aspects of disease management and, above all, a deeper understanding of the patient perspective, as patient satisfaction and clinical improvement represent the ultimate objective of any healthcare intervention.

The study sample comprised highly experienced vitreoretinal specialists practicing in public hospital settings, the majority of whom were male. This finding aligns with previously reported gender disparities documented among vitreoretinal surgeons in the literature [9]. Furthermore, most participants had established a specific clinical protocol for the management of patients with geographic atrophy (GA), in which optical coherence tomography (OCT), best-corrected visual acuity assessment, refraction, and fundus examination were included as essential diagnostic components in the majority of cases. This is consistent with the consensus definition for atrophy associated with age-related macular degeneration (AMD) proposed by Sadda et al. [10], which designated OCT as the reference standard and primary imaging modality for the diagnosis and staging of atrophy. Similarly, Kaiser et al. [8] reached an equivalent conclusion through the Geographic Atrophy Management Consensus (GA-MAC), developed using a Delphi panel methodology, establishing OCT as the preferred modality for both the diagnosis and longitudinal monitoring of GA. Additional imaging modalities proposed in consensus panels [10] as potentially valuable adjuncts for GA monitoring include fundus autofluorescence (FAF), near-infrared reflectance, and color fundus photography, all of which may provide complementary and confirmatory diagnostic information. Among the ophthalmologists surveyed in the present study, however, FAF was regarded as a relevant clinical investigation in the management of GA by only 33.3% of respondents, suggesting that this modality is considered complementary rather than essential, with OCT serving as the cornerstone of diagnostic practice. Finally, although deep learning algorithms applied to OCT imaging in AMD have demonstrated considerable promise over recent years, further prospective studies are warranted to establish their generalizability and clinical utility across diverse patient populations [11].

Regarding treatment recommendations among the surveyed ophthalmologists, these aligned with current consensus-based guidelines and indications [2,8,12,13,14]. In addition to appropriate optical correction to prevent visual loss attributable to refractive errors, the most frequently recommended interventions were nutritional supplements and low vision aids, particularly in cases of bilateral geographic atrophy (GA). Novel therapeutic approaches—including gene therapy, stem cell therapy, laser therapy, and recently FDA-approved intravitreal injections [13,14]—were also considered by a subset of the practitioners surveyed. Furthermore, the majority of surveyed ophthalmologists reported having a clearly defined patient follow-up protocol, comprising an initial visit within 3–6 months following treatment prescription and subsequent visits at 6–12 month intervals.

Consistent with the ophthalmologist group, the optometrist cohort comprised highly experienced professionals with a relatively balanced sex distribution. One area in which optometrists can contribute significantly to the care of GA patients is the prescription and fitting of low vision aids. In the present sample, all optometrists reported recommending low vision aids to improve visual performance; however, 71.4% indicated routinely prescribing them in their clinical practice. For the majority of optometrists, the timing of prescription was either upon completion of medical treatment or immediately following detection of the condition. This is consistent with three key recommendations for low vision referral previously established by a Delphi panel consensus in Australia: low vision referral should be based primarily on the impact of uncorrectable visual impairment on function and well-being; clinical measures of visual acuity and visual field should be regarded as secondary considerations; and patients should be fully informed about low vision services at an early stage of vision loss [15]. The most frequently prescribed low vision aids among the surveyed optometrists included magnifying glasses, zoom and text-resizing features on digital devices (both mobile and desktop), telescopes, microscopes, and selective absorption filters. These findings are also consistent with previously published reports on the most prescribed low vision aids across different regions of the world [16,17]. Emerging technologies, including augmented and virtual reality, have been introduced by several specialist practitioners, with growing evidence suggesting that the integration of these novel aids is becoming an established clinical reality [18].

Despite the generally appropriate recommendation of low vision aids, 12% of the surveyed optometrists did not recommend a visual rehabilitation program in all cases, thereby limiting the assurance that prescribed aids would be used effectively. Furthermore, 72.0% of optometrists recommended a visual rehabilitation program conducted by themselves, comprising in-office training sessions (lasting 20–40 min) combined with home-based visual training (lasting 10–30 min). Notably, the duration of visual rehabilitation programs varied considerably across professionals, underscoring the need for standardised, evidence-based rehabilitation protocols in the field of low vision. To date, only Coco-Martín et al. [19] have defined and validated a reading rehabilitation program for patients with AMD—developed in 2013—based on the principle of stepwise progressive goal achievement, whereby the difficulty of training tasks increases according to the level of success attained in preceding, less demanding tasks. Further research is warranted in this area, particularly incorporating the novel technologies and techniques emerging in the field of low vision.

It is noteworthy that the majority of optometrists performing visual rehabilitation programs reported familiarity with fixation re-education techniques [20] and compensatory saccadic eye movement training [21], yet were largely unaware of restoration techniques based on Gabor sinusoidal gratings or alternative visual stimuli, or of virtual reality-based training systems that have also demonstrated potential efficacy in improving visual performance in AMD patients [18,22,23]. Of additional relevance, most surveyed optometrists considered the field of low vision aid prescription and fitting in AMD to lack sufficient professional recognition and prestige, attributing primarily to the high cost of these services and the limited availability of adequately trained practitioners dedicated to this area. However, this perception may reflect a misconception among optometrists and could therefore be subject to bias, given that many patients have reported being uninformed about visual rehabilitation as a therapeutic option. In support of this, Lupón et al. [24] found in a prior cross-sectional study that 52% of the surveyed population had never heard of low vision services.

Finally, a cohort of patients was surveyed to capture the patient’s perspective. As a considerable proportion of respondents were unaware of their specific AMD subtype, patients across all AMD categories were included; consequently, conclusions regarding the patient perspective cannot be attributed exclusively to GA. Nevertheless, the data are of substantial value in understanding how patients perceive their clinical management and the information they effectively receive. Specifically, 35.1% of respondents reported having GA, while 32.4% were unaware of their exact AMD subtype and a further 32.4% had neovascular AMD. A considerable proportion of patients reported good visual acuity at the time of diagnosis, which had deteriorated by the time of survey completion. This trend did not differ significantly across AMD subtypes. Notably, in a previously published cross-sectional study, 77% of respondents agreed—either strongly or somewhat—that GA had impacted their vision more rapidly than anticipated [6]. In the present study, most patients reported a time interval from symptom onset to diagnosis ranging from 1 to 12 months, with ophthalmologists practicing in both public and private institutions being the primary professionals responsible for the initial evaluation. However, the ophthalmologist based in public hospitals was identified as the preferred professional for confirming the diagnosis and conducting subsequent patient follow-up. These patterns are consistent with those reported by Gupta et al. [7], who investigated the patient journey exclusively in a GA population. One notable discrepancy in relation to the ophthalmologist survey finding concerns treatment: most patients reported having received intravitreal injections as a form of treatment. It should be noted, however, that the patient sample included cases of neovascular AMD, and most GA patients indicated that they had initially received injections when their AMD was neovascular in nature and had subsequently progressed to a dry subtype with GA.

Among the cohort of patients surveyed, only 13.5% reported a diagnostic delay exceeding 24 months from initial symptom onset to confirmed diagnosis by a specialist. This subgroup consistently reported insufficient access to information, including a lack of guidance on low vision aids, minimal disease-specific education, and an absence of tailored recommendations for optimizing activities of daily living. Notably, none of these patients attended a private healthcare institution, potentially attributable to socioeconomic constraints. This finding underscores a persistent gap in the public healthcare system’s capacity to provide adequate and equitable care to all patients with age-related macular degeneration (AMD), highlighting the urgent need to optimize and standardize clinical protocols in the future.

One of the most notable findings was the significant discrepancy between the information that ophthalmologists and optometrists reported having provided to patients and patients’ own perception of the information they had received. Although most healthcare professionals confirmed providing foundational information covering the disease, its progression, treatment options, impact on quality of life, low vision aids, and patient associations, very few patients reported having received such information. This discrepancy may be primarily attributed to an ineffective communication process, stemming from time constraints during clinical consultations or the use of technical terminology not readily understood by patients. It should also be noted that most surveyed patients confirmed having been managed by the ophthalmologists and optometrists participating in the other two surveys. Scheffer et al. [25] reported current deficits in information provision for individuals with AMD, with patients frequently being inadequately informed regarding the chronic nature of the condition, treatment duration, nutritional considerations, and low vision rehabilitation options. Future research should investigate methods of optimising patient–healthcare professional communication to establish effective models, given the considerable impact of this issue on patients’ perception and experience of the disease. One potential criticism that could be raised here is that this discrepancy may be influenced by patient-related factors such as age, cognitive function, or health literacy. However, although these factors may have introduced some variability, their impact appears to be quite limited: it is impossible to complete a form if a patient is illiterate, and no patients with neurodegenerative disorders were identified within the sample. In any case, this cannot be considered an excuse to accept this limitation in the information-provision process, as it should be as efficacious as possible, regardless of the conditions under which it takes place. Furthermore, despite ophthalmologists and optometrists in the present sample also reporting that they inform patients about the availability of psychological and social support services, only 10.8% and 8.1% of patients reported having received such information, respectively. Gouliopoulos et al. [26] confirmed that AMD is associated with elevated rates of depression, highlighting the need for integrated ophthalmological and mental health care. A further noteworthy finding is that most patients reported receiving primarily two recommendations for improving daily well-being: the consistent use of sunglasses in sun-exposed environments and the monitoring of macular status using a grid-based self-assessment tool (the Amsler grid). This further underscores the need for more comprehensive and tailored recommendations capable of enhancing patients’ daily functioning and overall quality of life. No significant differences in any of these aspects concerning information and recommendations received were found among patients with neovascular AMD, GA, or unspecified AMD, confirming that these represent general findings potentially extrapolable to the broader AMD patient population.

Another relevant difference between healthcare professionals and patients pertains to the recommendation of low vision aids: whereas most professionals reported recommending such aids, only 5.4% of patients reported having received such a recommendation or prescription. This discrepancy further underscores the need for more effective patient–provider communication. This is of particular importance among patients with GA, who reported significantly greater difficulty with reading television subtitles and performing manual tasks. Nevertheless, certain activities of daily living were reported as equally challenging across all patient groups, including reading product prices while shopping, reading television subtitles, and reading newspaper text. These findings agree with previous studies demonstrating that patients with AMD experience increasing difficulty performing vision-related activities, with well-documented consequences for mental health [6,7]. Furthermore, given that considerable difficulties persisted following treatment prescription, a substantial proportion of patients (37.8%) reported no clinical improvement throughout the therapeutic process, with 45.9% expressing dissatisfaction with treatment outcomes.

This study has several limitations that warrant acknowledgement. First, although the sample size may be considered limited, the response rate was notably low across all three groups evaluated, falling below 30%. Nevertheless, the present findings may be regarded as preliminary, providing a foundation upon which future studies can be designed and oriented. Future studies must be performed with larger sample sizes and with equal representation of all health sectors from Spain in order to obtain more generalizable outcomes. For this reason, this study can be considered as a preliminary exploratory study. Second, the use of open-ended questions in certain sections, arising from the qualitative nature of the questionnaire, may constitute further limitation. The incorporation of standardised grading scales, supplemented by qualitative commentary fields for each item, may have yielded more quantifiable and meaningful results; this methodological consideration should be addressed in future investigations. In addition, it should be noted that the survey used was not a previously and strictly validated questionnaire; however, it should also be considered that no similarly comprehensive, previously validated survey on GA was available. Only a pre-validation study in terms of content validity was performed, with subgroups of 10 subjects for each group. One potential improvement for future research would be to expand this study by including a larger sample size, along with a more consistent reformulation of the survey that considers all the aspects identified during this preliminary experience. Third, the use of Google Forms as the data collection platform may also be regarded as a potential limitation. However, it should be noted that the survey was distributed exclusively within closed groups, with multiple responses prevented through individual email address verification, thereby reducing response bias. Moreover, the observed response rate was consistent with the characteristics of the target sample, given that the study was advertised solely in restricted settings. Finally, the questionnaire could have incorporated additional items to assess other relevant dimensions, such as adherence to prescribed treatments—particularly the use of low vision aids and participation in visual rehabilitation programmes—as well as the reasons underlying the limited adoption of emerging technologies in this field. These aspects warrant further investigation in future studies.

## 5. Conclusions

In conclusion, ophthalmologists specializing in retinal diseases in Spain appear to follow common clinical guidelines and treatment recommendations in the management of GA; however, the effectiveness of patient–provider communication requires improvement, as patients do not consistently share the same perception of the information and recommendations conveyed. Optometrists in Spain collaborate with ophthalmologists in the performance of diagnostic tests, the provision of disease-specific information, the recommendation and fitting of low vision aids, and the delivery of visual rehabilitation programmes to facilitate adaptation to such aids. Nevertheless, it seems that there are some potential limitations in the efficacy of communication between patients and healthcare professionals that should be investigated further. Furthermore, this study reinforces existing evidence on the substantial burden experienced by individuals living with GA, demonstrating the considerable impact that this condition can exert on health-related quality of life. The results of this preliminary study in Spain cannot be extrapolated to other countries in which other trends can be present and the professional competences of healthcare professionals can differ.

## Figures and Tables

**Table 1:** Surveys administered in the present study for ophthalmologists, optometrists, and patients. GA, geographic atrophy; ARMD, age-related macular degeneration.

Ophthalmologists	Optometrists	Patients
Section 1: Characterization of the participant How many years of professional experience do you have in the field of visual health? Sex Which setting best describes your current workplace?		Section 1: Characterization of the participant Gender Sex What is your current professional situation? Do you live in a rural environment or in a city?
Section 2 Clinical practice with GA patients Do you manage patients with GA in your clinical practice? Do you have a specific working protocol defined for this type of patient? Which diagnostic test or tests do you consider essential for GA? Once the presence of GA has been established (assuming that the presence of cataract has been previously ruled out), what would be your first treatment option in the case of unilateral disease? And in the case of bilateral disease? Once the initial treatment has been prescribed to the patient, when do you schedule the first follow-up visit? Following the first follow-up visit, how frequently do you schedule subsequent follow-up visits if the treatment is progressing satisfactorily? Once the presence of GA has been established and a significant cataract has been identified, what would be your intraocular lens implant option in the case of unilateral GA? And in case of bilateral GA? Which protocol until discharge after cataract surgery in cases of GA best corresponds to the one followed in your clinical practice? What type of information do you provide to the patient upon diagnosing the presence of GA? Do you recommend psychological support for patients with GA? Do you recommend social worker services for patients with GA in order to facilitate access to support aids and services of various kinds?	Section 2 Clinical practice with GA patients Do you help managing patients with GA in your clinical practice? Does your institution have a specific working protocol defined for this type of patient? Which diagnostic test or tests do you consider essential for GA? Which of the treatments for GA are you familiar with? Within your scope of practice and your competences, what is your role in the management of patients with GA? What type of information do you provide to the patient upon diagnosing the ophthalmologist the presence of GA? Do you recommend psychological support for patients with GA? Do you recommend social worker services for patients with GA in order to facilitate access to support aids and services of various kinds?	Section 2 What do you know about your ARMD? Do you know what type of ARMD you have? Which eye is affected? Do you wear optical correction in glasses or contact lenses? How would you rate the vision you currently have with your glasses or contact lenses? How would you rate the vision you had with your glasses or contact lenses when you were diagnosed with ARMD? What type of symptoms do you currently experience? How long did it approximately take from when you started noticing symptoms to the definitive diagnosis of ARMD? Who was the first professional you went to consult about what was happening to you? Which professional confirmed the diagnosis of ARMD exactly? Once you were given the diagnosis of ARMD, what was the treatment you were indicated? In the case of receiving intraocular or intravitreal injections, how many have you received to date? How do you consider the improvement of your vision with the indicated treatment? Which healthcare professional is monitoring how your vision is evolving? Has your ARMD treatment been modified as time has gone by? What other treatments were you indicated? What was the reason for the change in treatment? How long did it approximately take from the initial treatment to the change of treatment? Are you satisfied with the treatments that have been provided to you? If not, why?
Section 3 Low vision aids Do you recommend the use of low vision aids for GA? If not, what is the main reason for not recommending them?	Section 3 Low vision aids Do you recommend the use of low vision aids for GA? If not, what is the main reason for not recommending them? Do you prescribe and fit low vision aids for patients with GA? If answered affirmatively, how many patients have you fitted with low vision aids? At what stage do you initiate the fitting of low vision aids? What types of low vision aids do you prescribe? Do you consider that the field of prescribing and fitting low vision aids in GA has recognition and prestige? What do you consider to be the main factors that could influence the fact that more low vision aids are not recommended or prescribed in GA? Do you think there should be a regulated university specialization that qualifies those professionals who want to dedicate themselves to low vision?	Section 3 Use of low vision aids Have you been recommended the use of any low vision optical aid? In the case of having been prescribed a low vision aid, have you undergone a visual rehabilitation program with the prescribed aids?
Section 4 Telescopic intraocular lenses (IOLs) Do you have any experience with the implantation of telescopic IOLs in GA? If so, how many patients have you implanted? How would you describe your experience with telescopic lenses? In the event of IOL implantation, have you recommended a visual rehabilitation programme following the procedure?	Section 4 Visual rehabilitation with low vision aids Do you recommend a visual rehabilitation programme alongside the prescribed low vision aids? What is the usual average duration of such a visual rehabilitation programme? What does the visual rehabilitation program consist of in your clinical practice? On average, how many minutes do the visual rehabilitation sessions you carry out in your practice last? On average, how many minutes do the visual rehabilitation sessions you prescribe for home last? In the field of visual rehabilitation, which training procedures do you use?	Section 4 Information and clinical support received What type of information have you received from the healthcare professional about ARMD? What type of healthcare professional has provided you with the information? Have you been recommended any type of psychological support? Have you been recommended the services of social workers in order to facilitate aids or support services of different kinds? Were you aware that there are telescopes that can be implanted inside the eye to improve vision in patients with ARMD? Which of the general recommendations for people with low vision, in order to help them manage more easily in their daily environment, were you aware of? How many visits have you made to the ophthalmologist in the last year? In what situations do you currently experience difficulty in your daily life?
	Section 5 Telescopic intraocular lenses (IOLs) Are you familiar with the treatment using telescopic intraocular lens implants in GA? Would you recommend telescopic IOL implants in GA?	

**Table 2:** Recommendations provided by healthcare professionals as reported by patients, and symptoms and difficulties described by patients.

Description	% of Patients	Number of Patients
Patient’s symptoms		
Generalized blurred vision Blurred vision restricted to the central visual field Absence of central visual field vision Distortion of perceived images Altered image size perception Perception of lights or sudden flashes Difficulty in color discrimination Frequent glare sensation Difficulty in reading	48.6% 18.9% 27.0% 32.4% 5.4% 24.3% 21.6% 37.8% 73.0%	18 7 10 12 2 9 8 14 27
Current limitations and difficulties in activities of daily living		
Reading newspaper text Viewing messages on WhatsApp on a mobile device Recognizing the faces of familiar individuals Reading product prices when shopping Walking on uneven surfaces (e.g., cobblestone pavements) Reading subtitles on television Performing manual tasks (e.g., carpentry work) Engaging in activities or hobbies of personal interest	64.9% 48.6% 54.1% 67.6% 43.2% 67.6% 32.4% 37.8%	24 18 20 25 16 25 12 14
Recommendations received		
Consistent use of sunglasses in sun-exposed environments Monitoring macular status by means of a grid-based self-assessment tool (known as the Amsler grid) Monitoring disease progression by tracking increasing difficulty in reading Maintaining household furniture and utensils in a consistent, fixed location Marking door and window frames and edges with high-contrast colors Marking the edges of staircases, handrails, and steps Using colored tableware and kitchen utensils to enhance visual contrast Preferential use of ceramic hob or induction cookers. When cooking with gas, avoiding the use of matches and opting for a lighter instead Avoiding mirrors with reflected light that may cause glare Avoiding glossy tiles in bathrooms and kitchens Creating shaded environments by means of blinds or sheer curtains Watching television without ambient lighting in the room Placing signs at eye level Using visors Using lined paper to maintain orientation when reading or writing Writing with black, broad-tipped markers	83.8% 64.9% 29.7% 16.2% 8.1% 10.8% 13.5% 8.1% 10.8% 5.4% 16.2% 2.7% 2.7% 21.6% 13.5% 13.5%	31 24 11 6 3 4 5 3 4 2 6 1 1 8 5 5

## Data Availability

Data are available from the corresponding author, D.P.P., upon reasonable request.
